# Structural and Biochemical Characterization of Apicomplexan Inorganic Pyrophosphatases

**DOI:** 10.1038/s41598-017-05234-y

**Published:** 2017-07-12

**Authors:** Abhishek Jamwal, Manickam Yogavel, Malik. Z. Abdin, Swatantra. K. Jain, Amit Sharma

**Affiliations:** 1 Molecular Medicine Group, International Centre for Genetic Engineering and Biotechnology, Aruna Asaf Ali Marg, New Delhi, 110067 India; 2 Department of Biotechnology, Jamia Hamdard, New Delhi, 110063 India; 3 Department of Biochemistry, Hamdard Institute of Medical Sciences, 110063 New Delhi, India

## Abstract

Inorganic pyrophosphatases (PPase) participate in energy cycling and they are essential for growth and survival of organisms. Here we report extensive structural and functional characterization of soluble PPases from the human parasites *Plasmodium falciparum* (PfPPase) and *Toxoplasma gondii* (TgPPase). Our results show that PfPPase is a cytosolic enzyme whose gene expression is upregulated during parasite asexual stages. Cambialistic PfPPase actively hydrolyzes linear short chain polyphosphates like PP_i_, polyP_3_ and ATP in the presence of Zn^2+^. A remarkable new feature of PfPPase is the low complexity asparagine-rich N-terminal region that mediates its dimerization. Deletion of N-region has an unexpected and substantial effect on the stability of PfPPase domain, resulting in aggregation and significant loss of enzyme activity. Significantly, the crystal structures of PfPPase and TgPPase reveal unusual and unprecedented dimeric organizations and provide new fundamental insights into the variety of oligomeric assemblies possible in eukaryotic inorganic PPases.

## Introduction

Protozoan parasites from phylum Apicomplexa cause substantial morbidity and mortality worldwide. The most widely studied of these parasites are the Plasmodium species - causative agents of malaria. In 2015, there were ~214 million new cases of malaria and ~0.25 million deaths due to the malaria parasite *Plasmodium*, while the closely related parasite *Toxoplasma gondii* (*T*. *gondii*) infected 25% of world’s population^1, 2^. The most virulent human malaria parasite is *P*. *falciparum*, and like other human infecting plasmodia, it resides and develops inside erythrocytes during asexual stages leading to clinical symptoms associated with malaria. Intra-erythrocyte development of *P*. *falciparum* is a complex and multistage process in which development proceeds via rings to the trophozoite phase of nutrient acquisition and then to the multiplicative schizont phase^3^. The ability of malaria parasites to cause disease is dependent on their growth inside erythrocytes. Therefore, asexual stages have been targeted for developing therapeutics, and most anti-malarial kill blood stage parasites. However, fast-spreading drug resistance is a major problem in malaria treatment, and it is slowly rendering malaria drugs ineffective^4^. Therefore, it is ever more important to continually investigate basic malaria parasite biology so as to lay foundations for targeting its bimolecular machinery with new drugs.

The enzyme inorganic pyrophosphatase (PPase) catalyzes the hydrolysis of pyrophosphate (PP_i_) to inorganic phosphate (P_i_). This is an exergonic reaction and can be coupled to several unfavorable and energy demanding biochemical transformations such as DNA replication, protein synthesis and lipid metabolism^5^. PPases include membrane associated V-H^+^-PPases (vacuolar H^+^-translocating PPases) and soluble form PPases, where latter comprise two families that differ in their sequence and structure^6^. Family I PPases are Mg^2+^ dependent enzymes known to exist as homo-hexamers in prokaryotes and dimers in eukaryotes^6^. Family II PPases are Mn^2+^ dependent enzymes with bi-domain structures, and active in dimeric or trimeric forms^7^.

Apart from the cytoplasm, PP_i_ is also present in acidocalcisomes – these are organelles enriched in polyphosphates (polyP) and cations – these are acidified by proton pumps^8^. In acidocalcisomes, PP_i_ is generated from hydrolysis of polyphosphates (polyP). Vacuolar Soluble Protein (VSP) is a type I acidocalcisomal PPase according to the divalent metal cofactor used - Mg^2+^ or Zn^2+^ ^9^. VSP specifically hydrolyzes either PP_i_ or polyP^9^. Also, VSP1 plays a critical role in *T*. *cruzi* persistence inside its host by maintaining osmotic balance of parasites via regulating the phosphate content in acidocalcisomes^10^. The absence of a soluble PPase would lead to the build-up of toxic levels of PP_i_, accounting for the essential nature of this enzyme. In *C*. *elegans*, a null mutant of PPase was developmentally arrested at the larval stage with defects in intestinal morphology^11^. Mutant PPases were also found to be associated with cell cycle arrest and cell death in fermenting yeast^12^. Increased expression and activity of cytosolic PPase has been linked with aging in rat and mouse^13^. In humans, over-expression of cytosolic PPase is associated with many types of cancer such as those of breast and lung, ovarian, and hepato-carcinoma^14–17^.

Due to their essential roles in metabolism, PPases have been studied as potential drug targets with a focus on pathogenic organisms. For example, a novel series of anti-PPase small molecules were shown to target drug resistant strains of *Staphylococcus aureus* ^18^. In another study, selective inhibition of short-chain polyP activity of VSP1 by small molecule inhibitors provided protection against *T*. *brucei* infection in a mouse model^19^. Furthermore, a distinct allosteric site has been exploited to target *M*. *tuberculosis* PPases^20^. These studies show that PPases can be targeted at multiple structural levels, and offer hope of obtaining inhibitors by utilizing distinct structural and functional properties of PPases. Recently, our group elucidated the atomic structure of *T*. *brucei* VSP1 and highlighted several of its distinct features that may have implications for inhibitor design^21^. The soluble PPase from *Toxoplasma gondii* (TgPPase) has also been studied biochemically in the past^22^.

In present study, we focused on a previously uncharacterized *P*. *falciparum* soluble inorganic pyrophosphatase (PF3D7_0316300.1) referred to as PfPPase from hereon. Comparative sequence and domain analysis data suggested that PfPPase consists of 380 amino acids and differs markedly from homologous enzymes in its N-terminal region that is extended by ~76 amino acid rich in asparagines (~30% of the region), a feature often associated with low-complexity regions in *P. falciparum* ^23^. In contrast, the N-terminal region of TgPPase (residue 1–78) is rich in glycine and serine residues. We report structural and biochemical characterization of these two apicomplexan PPases. We also present insights into the dimerization modes of eukaryotic family I soluble PPases.

## Results and Discussion

### Characterization of *P*. *falciparum* PPase expression levels

**Figure 1:**
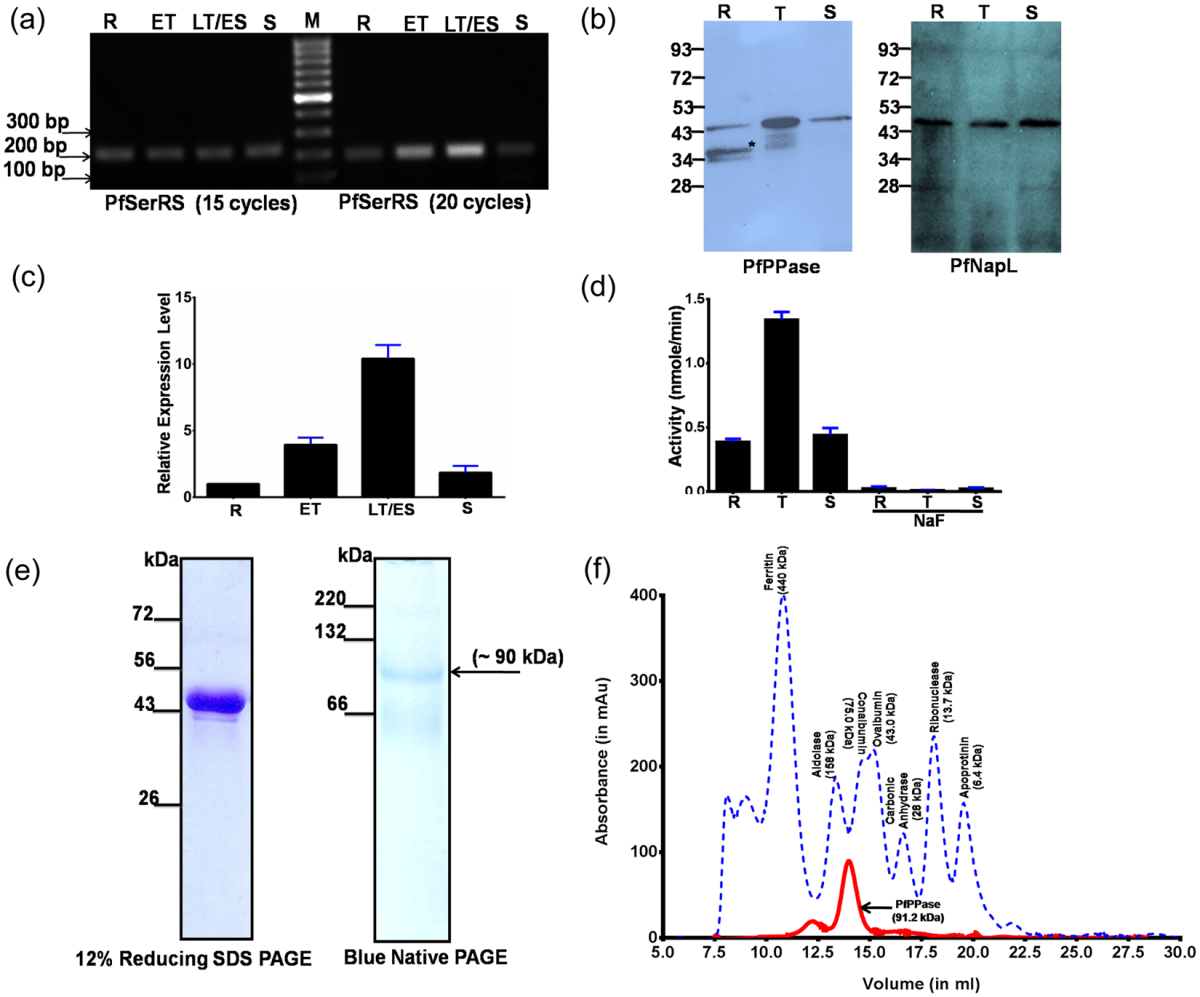
Expression of PfPPase. (**a**) Semi-quantitative reverse transcription (RT)-PCR analysis for PfPPase gene with seryl-tRNA synthetase gene as a gel loading control. The number of RT-PCR cycles is shown in the brackets, (**b**) Western blot analyses of PfPPase expression analysis in *P*. *falciparum* intra-erythrocyte stages. The sorbitol synchronized parasite lysate was resolved on 12% SDS–PAGE and subjected to western blotting using anti-PfPPase antibodies (left panel). Additional band (asterisk) of size ~35 kDa is seen in the ring stages. To confirm equal amount of proteins in parasite lysate the blot was probed with anti-PfNapL antibodies (right panel). (**c**) The relative expression level of each PfPPase gene quantified by real-time quantitative RT-PCR. R, rings; ET, early trophozoites; LT, late trophozoites; S, schizonts. (**d**) Total PPase activity in cell lysate was inhibited by sodium fluoride. (**e**) Purified recombinant PfPPase from *E*. *coli* analyzed by SDS-PAGE and blue native PAGE. (**f**) Size exclusion chromatography elution profile/peaks of full-length PfPPase monitored by absorbance at 280 nm. ﻿﻿Elut﻿ion﻿ profile of ﻿standard﻿ M_wt_ markers is shown in dashed lines colored in blue.

Reverse transcription PCR-based (RT-PCR) expression profile of the gene corresponding to *PfPPase* (PF3D7_0316300.1) suggested that PfPPase was transcribed during all asexual stages of *P*. *falciparum*; its expression increased relative to ring stage, during late trophozoite (LT)/early schizont (ES) and reduced again when schizonts mature (Fig. 1a). This gene expression profile was complimented by western blotting data that showed protein expression during all three stages but with higher expression during the trophozoite stage (Fig. 1b). Our quantitative PCR (qPCR) analyses using threshold C_T_ supported our semi-quantitative PCR and western blotting profiles ﻿(Fig. 1c). The qPCR data suggested that expression of PfPPase increased ~4 fold as parasite rings transformed into ﻿ET﻿ (Fig. 1c). Interestingly, maturation of ET into LT/ES was accompanied by further increase in expression, which was ~10 fold higher relative to in rings (Fig. 1c). Eventually, the expression was restored to near basal levels in mature schizonts (Fig. 1c). Therefore, our results indicated that PfPPase levels were differentially regulated during asexual stages of *P*. *falciparum*, and stimulation of PfPPase expression could be attributed to anabolic nature of LT/ES stages that display high rates of protein synthesis and DNA replication.

The cell extracts derived from different *P*. *falciparum* asexual stages actively hydrolyzed PP_i_ in presence of 1 mM Mg^2+^ (Fig. 1d). In addition, this activity was inhibited completely by 1 mM NaF, a known inhibitor of PPase activity (Fig. 1d). These results further supported our gene/protein expression data that indicated presence of a functional PPase in *P*. *falciparum*. For functional and structural analysis of PfPPase, the enzyme was over-expressed and purified from *E*. *coli*. SDS-PAGE analysis of PfPPase confirmed its theoretical molecular weight (M_wt_) ~45 kDa (Fig. 1e). However, the trace on gel filtration chromatogram showed that PfPPase existed predominantly as a dimer in solution with a minor tetrameric peak (Fig. 1f).

### PfPPase is a cytosolic enzyme

**Figure 2:**
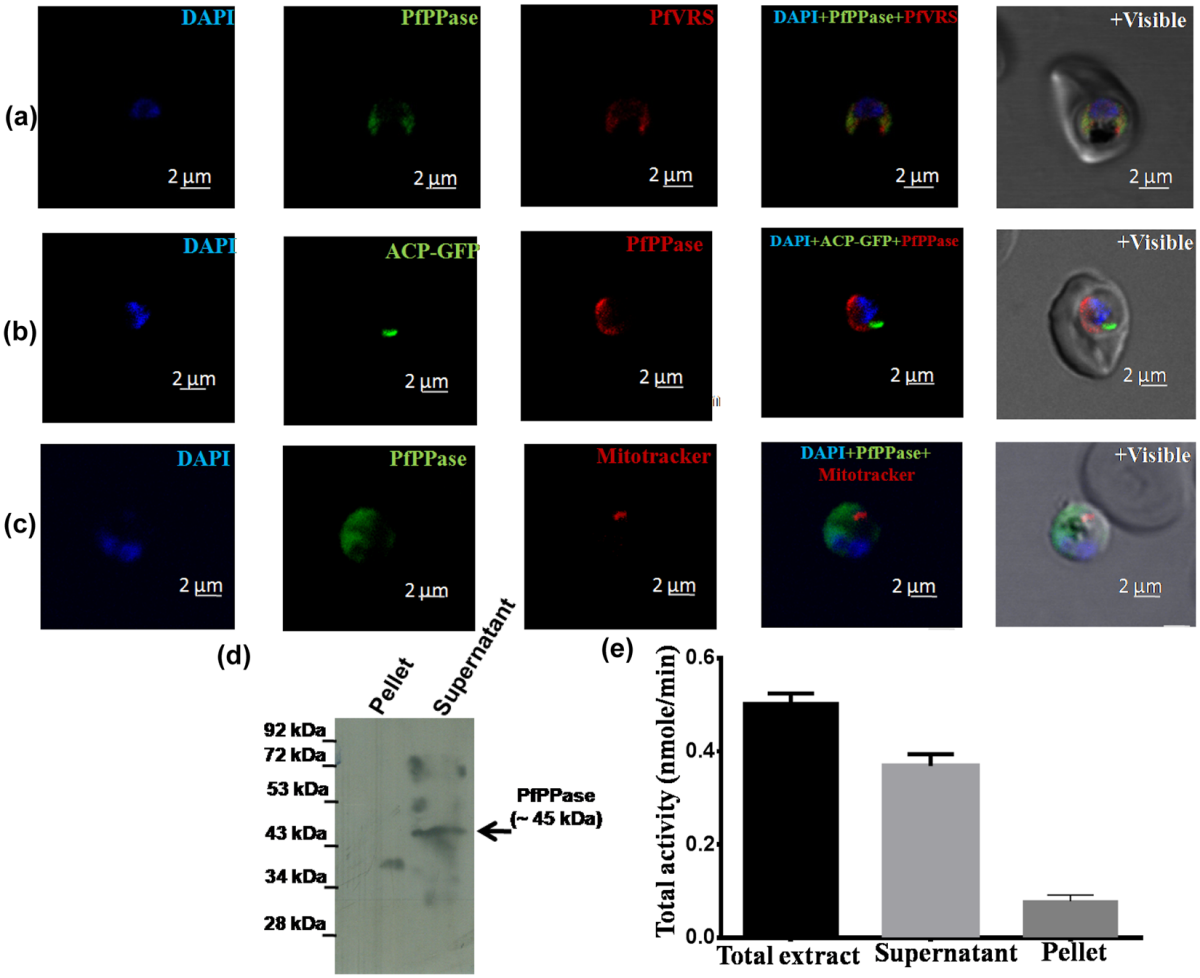
PfPPase localizes to cytosol. (**a**) Fluorescent staining of *P*. *falciparum* trophozoite cells using anti-PfPPase antibody (Alexa 488, green). It is apparent that PfPPase co-stains with cytosolic protein marker Pf Ed-VRS(Alexa 594, red) (**b**) and (**c**) note the non-apicoplast and non-mitochondrial localization of PfPPase where apicoplast is stained green (D10-ACP-GFP) and mitochondria is stained red (Mitotracker), (**d**) Western blot analysis of subcellular fractions of *P*. *falciparum* trophozoite on 12% SDS-PAGE. Arrow indicates monomeric PfPPase size (~45 kDa) in cell lysate. Equal protein amounts (40 μg) from supernatant (S) and pellet (P) fractions were loaded. The molecular mass standards (in kDa) are shown on the left-hand side (**e**) PPase activity in lysate, supernatant and pellet fractions.

To investigate localization of PfPPase in asexual stages of malaria parasites, we performed indirect immunoflorescence assays (IFA) using antibodies against purified recombinant PfPPase protein and a previously described protocol^24^. As evidenced by co-localization of Pf-Ed-VRS (valine–tRNA synthetase, a known cytosolic maker), the endogenous localization of the PfPPase enzyme was cytosolic (Fig. 2a). In addition to the cytosol, IFA studies with organelle markers (acyl-carrier protein-green fluorescent protein and mitotracker) revealed that PfPPase was not localized either in the apicoplast or the mitochondria (Fig. 2b and c) in asexual parasite stages. Western blot analysis of cytosolic and membrane fractions of trophozoites further confirmed cytosolic localization of PfPPase (Fig. 2d). Consistent with this, the total activity of the enzyme was predominantly in the soluble fraction obtained from parasites (Fig. 2e).

### Kinetic analysis of PfPPase and substrate binding

**Figure 3:**
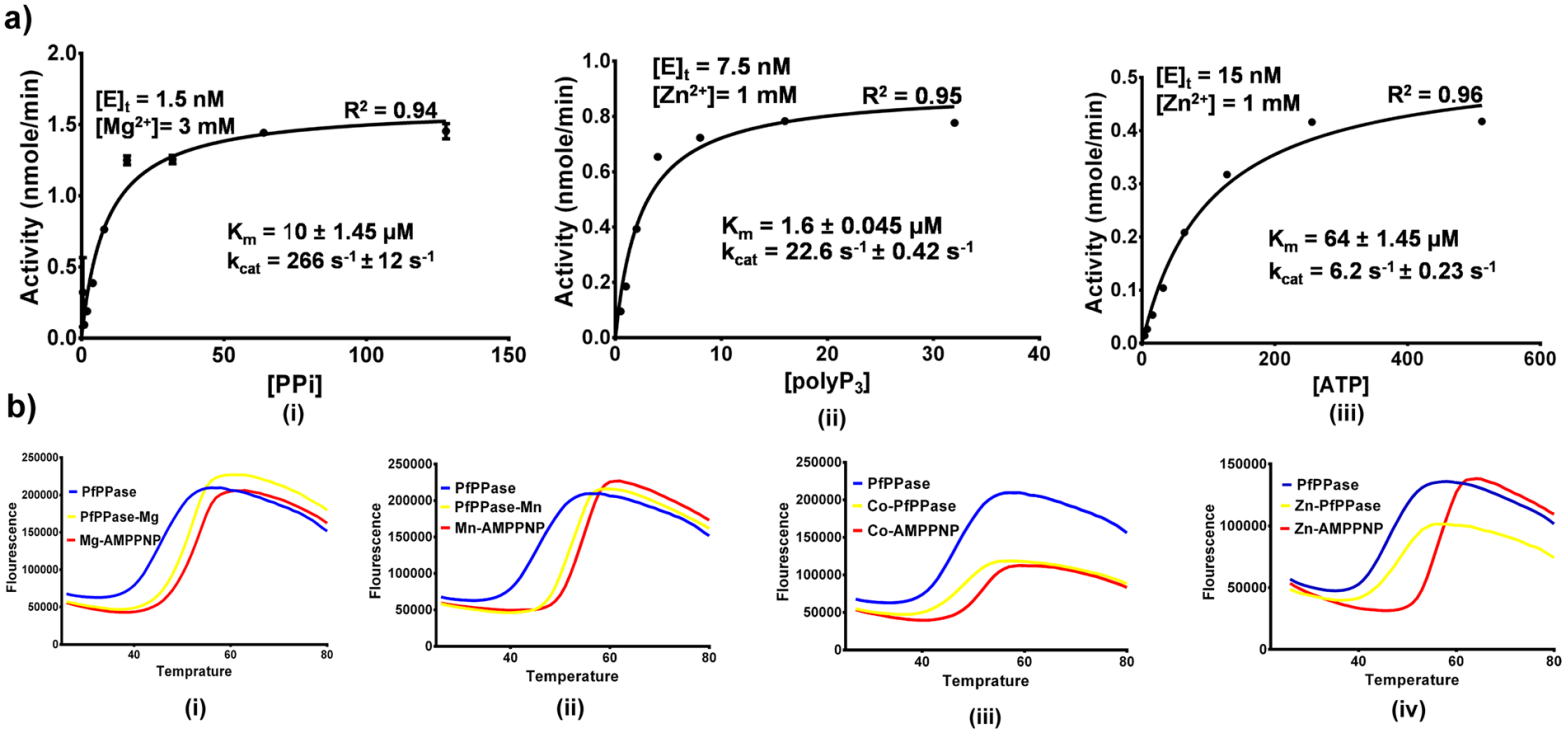
Substrate specificity of PfPPase. (**a**) Kinetic analysis of PfPPase. Michaelis–Menten kinetics of PfPPase was assessed for each of the three substrates (i) PP_i_ (ii) polyP_3_ (iii) ATP. (**b**) Thermal Stability curves of PfPPase in a ternary complex with AMPPNP (i) Mg^2+^ (ii) Mn^2+^ (iii) Co^2+^ (iv) Zn^2+^.

We examined PfPPase activity using a colorimetric assay for phosphate estimation^25^. PfPPase was found to be capable of utilizing PP_i_, polyP_3_ and ATP as substrates (Fig. 3a,i–iii). PP_i_ hydrolysis by PfPPase showed an absolute requirement for divalent cations and had a turnover number (k_cat_) of 266 s^−1^ at 37 °C and optimum pH of 7.2 in presence of 3 mM Mg^2+^ (Fig. 3a,i). Other divalent cations such as Co^2+^, Zn^2+^ and Mn^2+^ stimulated PP_i_ hydrolysis but with lower efficiency (Table 1). The relative PfPPase PP_i_ activity conferred by divalent metal ions fell in the order Mg^2+^ > Co^2+^ ~ Zn^2+^ > Mn^2+^. However, Zn^2+^ was the preferred co-factor for hydrolysis of polyP_3_ and ATP (Table 2). PfPPase displayed a K_m_ of ~64 µM for ATP and it is noteworthy that the intracellular concentration of ATP in *P*. *falciparum* is in milimolar range, which thus suggests physiological significance of the above K_m_^26^. Overall; these data highlight the cambialistic properties of PfPPase. However, it is clear from k_cat_/K_m_ values that the catalytic efficiency of PP_i_ hydrolysis is higher than for other substrates (Tables 1 and 2). Surprisingly, Mg^2+^ failed to stimulate hydrolysis of both polyP_3_ and ATP. In a previous study Zyryanov et. al., had shown that the rate determining step in ATP hydrolysis was breakage of the P-O bond by PPases from *S*. *cerevisae*, *E*. *coli*, *S*. *mutans* and rat liver^27^. They further showed that higher efficiency of transition metal ions compared with Mg^2+^ emanated from stronger binding of the terminal phosphate of ATP or polyP_3_ in presence of the transition metal, which allowed more favorable position for catalysis^27^. We probed this idea using protein thermal shift (PTS) assays that provide an assessment of the stability of protein and protein-ligand complexes based on melting temperature (T_m_)^28^. PTS analysis revealed substantial difference in AMPPNP (a substrate analog of ATP) binding in presence of transition metals and Mg^2+^ ions. As shown in Table 3 and Fig. 3b(i–iv), the T_m_ shifts suggested a higher affinity for Zn^2+^-AMPPNP (ΔT_m_ = 10.3 °C) than Mg^2+^-AMPPNP (ΔT_m_ = 0.8 °C). Moreover, the order of T_m_ shift with AMPPPNP was same as the order of catalytic efficiency of ATP i.e. Zn^2+^ > Co^2+^ > Mn^2+^ > Mg^2+^. These results thus validate and reaffirm the link between catalytic efficiency and strong binding of ATP or polyP_3_ in presence of transition metals.

**Table 1:** Kinetic parameters of PP_i_ hydrolysis by PfPPase in presence of transition divalent cations.

Cofactor	Concentration (mM)	pH	K_m_ (µM)	k_cat_ (s^−1^)	k_cat_/K_m_(M^−1^s^−1^)
Zn^2+^	1	7.2	22.6 ± 4.3	8.3 ± 1.2	3.67 × 10^5^
Co^2+^	1	7.6	19.4 ± 4.1	24.3 ± 4.3	1.36 × 10^6^
Mn^2+^	1	7.6	92.8 ± 4.9	6.3 ± 1.4	0.67 × 10^5^

**Table 2:** Kinetic parameters of polyP_3_ and ATP hydrolysis by PfPPase in presence of transition divalent cations.

Cofactor	Concentration (mM)	pH		k_cat_(s^−1^)		K_m_ (µM)		k_cat_/K_m_ (M^−1^s^−1^)	
		polyP_3_	ATP	polyP_3_	ATP	polyP_3_	ATP	polyP_3_	ATP
Zn^2+^	1	7.0	7.2	26.3 ± 3	6.3 ± 1.2	1.6 ± 0.3	64 ± 4.2	1.6 × 10^7^	0.9 × 10^5^
Co^2+^	1	7.4	7.2	9.6 ± 2.1	2.8 ± 0.6	12.6 ± 1.4	96 ± 7.8	7.6 × 10^6^	0.2 × 10^5^
Mn^2+^	1	7.2	7.2	1.6 ± 0.7	0.8 ± 0.1	8.5 ± 2.8	180 ± 21.9	1.8 × 10^5^	0.8 × 10^4^

**Table 3:** Melting temperature (T_m_) of PfPPase with divalent cations and AMPPNP.

Experiment	T_m_ (°C)	Experiment	T_m_ (°C)	ΔT_m_ (°C)	ΔT_m_ (°C) (ΔT_m_ = T_m2_−T_m1_)
PPase (Apo)	46	PPase + AMPPNP	46.2	0.2	0.2
PPase + Mg^2+^ (1 mM)	53	PPase + AMPPNP + Mg^2+^	53.8	0.8	0.8
PPase + Mn^2+^ (1 mM)	54	PPase + AMPPNP + Mn^2+^	56.3	2.7	2.7
PPase + Zn^2+^ (1 mM)	48	PPase + AMPPNP + Zn^2+^	59.3	10.3	10.3
PPase + Co^2+^ (1 mM)	49	PPase + AMPPNP + Co^2+^	52.3	3.3	3.3

### Crystal structures of PfPPase and TgPPase

**Figure 4:**
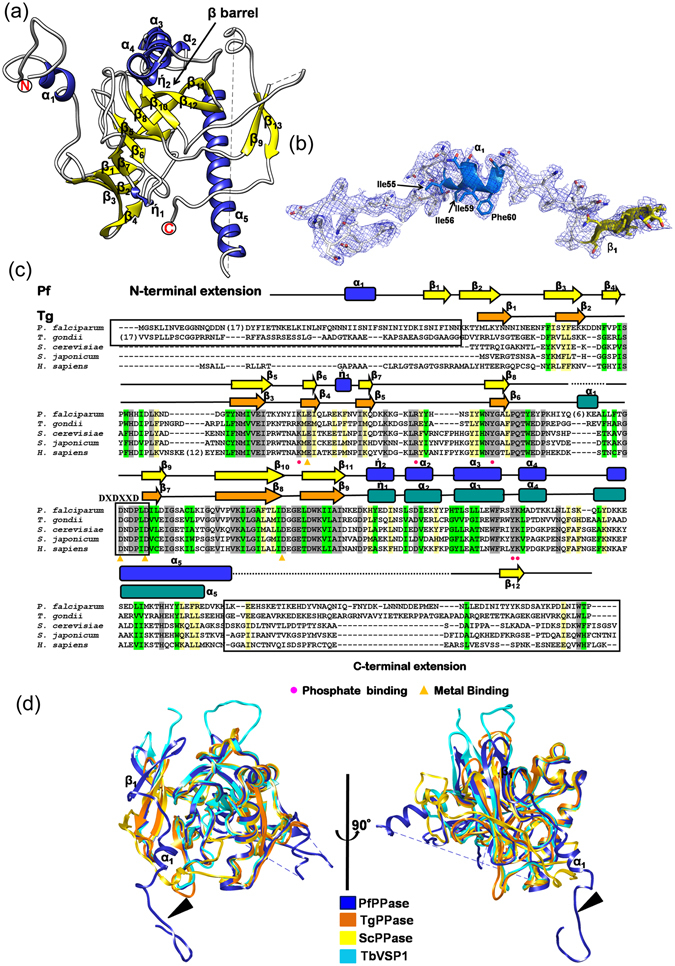
Crystal structure of PfPPase. (**a**) Monomeric structure of PfPPase shown in ribbon form. α-helices, β-strands and coils of PfPPase are highlighted in blue, yellow and white respectively; (**b**) 2F_o_-F_c_ map contoured at σ = 0.9 for PfPPase N-terminal region where electron density is shown as blue mesh. Residues involved in dimer formation are shown as sticks; (**c**) Sequence alignment of PPases colored by residue conservation. Secondary structure elements (yellow arrow:β- sheet; blue rectangular boxes; α-helices); yellow triangles (pyrophosphate) and pink circles (metal binding) indicate active site residues; (**d**) Structural superposition of PfPPase onto TbVSP1, ScPPase, TgPPase reveals conservation of core structure, with differences in peripheral parts of the structure. N-terminal extension of PfPPase is indicated by arrow.

To obtain a comprehensive structural description of PfPPase and TgPPase we determined their crystal structures. We were successful in obtaining crystals of seleno-methionine (Se-Met) labeled PfPPase and hence used single wavelength anomalous (SAD) technique to obtain the crystal structure of PfPPase. The final structure was refined to highest resolution of 3.2 Å with R_work_/R_free_ of 0.22/0.27 (Table 4). Crystals of PfPPase belonged to space group C2 with five molecules in the asymmetric unit and Mathew’s coefficient of V_m_ ~ 3.0. Overall, the PfPPase crystal structure showed simple domain architecture, typified by five stranded β-barrel β_4_ and β_7_-β_10_ (Fig. 4a). This β barrel is flanked by helices α_3_ (residue; 264–280) and α_5_ (residue; 293–316). There are two 3_10_ helical turns η_1_and η_2_ at residues 144–146 and 258–251. The first 36 residues of PfPPase are missing in the current crystal structure and thus the structure extends from residues 37 to 380. The N-terminal region of PfPPase extends from 36–76 residues. This region is composed of a stretch that lacks secondary structure (residues 36–54), a small highly hydrophobic helical region (α_1_, residues 54–60) that is then followed by a strand (β_1_, residues 70–76) that leads into the PfPPase domain (Fig. 4b-c). Residues 324–352 are highly disordered showing no clear density in the crystal structure.

**Table 4:** Data collection and Refinement statistics.

	TgPPase-Mg^2+^	PfPPase-P_i_
**Data collection**		
Space group	P3_2_21	C2
a,b,c (Å)	89.26, 89.26, 159.71	253.19, 85.23, 108.45
α,β,γ (°)	90°, 90°, 120°	90°, 114.36°, 90°
R_meas_	0.06(1.48)	0.12(0.59)
R_pim_	0.03(0.46)	0.05(0.26)
R_merge_	0.05(0.1.41)	0.11(0.72)
I/σ(I)	38.30 (1.56)	38.1 (2.71)
CC_1/2_	1.00 (0.65)	0.99 (0.738)
Completeness	98.1 (96.0)	97.80 (99.70)
Redundancy	10.4 (8.9)	5.60 (5.40)
**Refinement**		
Resolution (Å)	43.6–2.35	44.9–3.2
Number of reflections	28397	33201
R_work_/R_free_	0.204/0.232	0.222/0.269
**B-factor (Å** ^**2**^)		
Protein	38.0	65.0
Ligand/ion	28.0	99.0
Water	32.00	—
**rmsd**		
Bond length (Å)	0.005	0.006
Bond Angle (°)	0.914	1.241
**Ramachandran**		
Preferred Regions	96.76	95.57
Allowed Regions	3.25	4.03
Outliers	0.0	0.40

We next determined the crystal structure of TgPPase using molecular replacement method using ScPPase (PDB ID = 1WGJ) as a template. The TgPPase crystal structure was refined to 2.35 Å and showed the same structural overall fold as PfPPase. However, TgPPase lacked both N- and C-terminal regions and the structure contains residues 74–308 (Table 4 and Supplementary Figure 1a), most likely due to proteolysis of N-terminal region during crystallization. Structural homology searches using DALI server with PfPPase and TgPPase indicated that both structures show high similarity to PPase domain of *T*. *brucei* VSP1 (TbVSP1 PDB: 5C5V; Z = 30) and ScPPase, PDB: 1WGJ; Z = 18). Subsequent structural comparisons revealed that architectural differences between PfPPase, TgPPase, ScPPase and TbVSP1 existed mostly in surface areas such as the connections or loops between helices and strands (Fig. 4d). An interesting and a key distinct feature of PfPPase is its N region (residues 36–76) that stretches away from the structural core and is absent in both *T*. *brucei* and *S*. *cerevisae* PPases (Fig. 4c-d). Interestingly, our data showed that deletion of the N-terminal region (residues 1–76) from PfPPase (ΔN-PfPPase) resulted in PPase aggregation and loss of enzyme activity (Supplementary Fig. 2a). Furthermore, the thermal stability of the ΔN-PfPPase could not be derived as the fluorescent probe (SYPRO orange) was already bound to the protein at room temperature, an indication that protein was likely already unstable (Supplementary Figure 2b).

### Active sites in PfPPase and TgPPase

**Figure 5:**
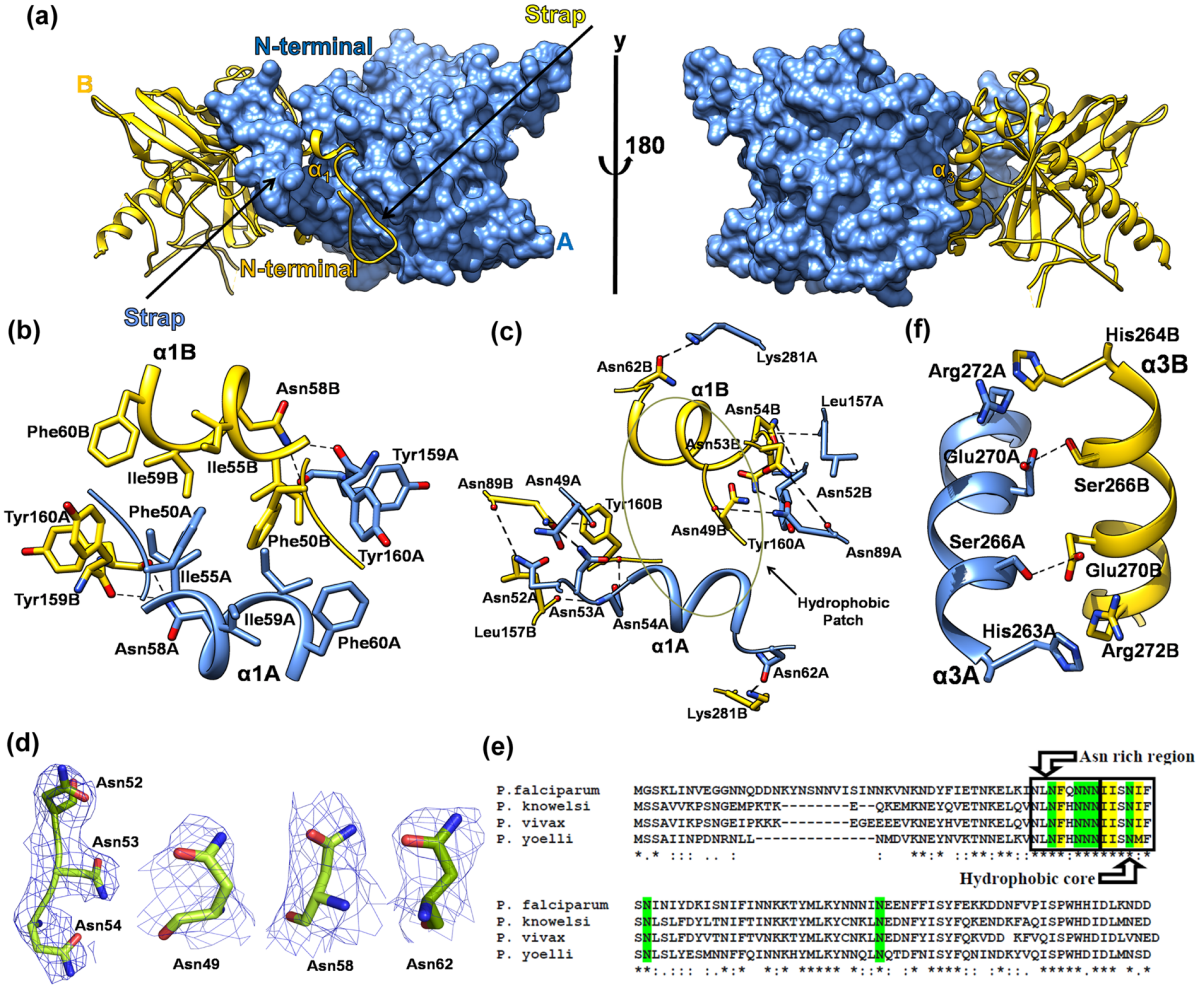
Dimeric assembly of PfPPase. (**a**) Two views of PfPPase dimer showing its strap-like N-terminus involved in dimer formation (**b**), (**c**) and (**f**) Inter-subunit contacts with important residues shown as sticks. (**d**) 2F_o_-F_c_ electron density maps contoured at σ = 1.0 showing clear densities for interfacial asparagines in the PfPPase structure. (**e**) Sequence alignment of plasmodial PPases shows conservation of N-region interfacial residues.

Structural comparisons of PfPPase and TgPPase with ScPPase/TbVSP1 revealed that residues responsible for binding of PP_i_ and of Mg^2+^ were located on the top of β-barrel and active site residues within were highly conserved (Supplementary Figure 3a–d and Fig. 4c). Crystals of PfPPase were grown in high concentration of PP_i_ and in presence of Mg^2+^ though we were unable to assign Mg^2+^. PfPPase active site showed electron density for only one P_i_ molecule in two (B and C) out of five subunits (A–E) in the asymmetric unit (Supplementary Figure 3e). In structural comparisons with TbVSP1 and ScPPase, this P_i_ molecule in PfPPase closely corresponded to the location of P_i_ molecule of the bound PP_i_ that is not directly attacked (P1) (Supplementary Figure 3a). This observation suggested that the directly attacked phosphate group (P2) of the PP_i_ was first to dissociate from the active site of PfPPase. In contrast to PfPPase, the active site of TgPPase contained two bound Mg^2+^ ions (Fig. 5b). One Mg^2+^ was bound at M1 site coordinated by Asp190, Asp195 and Asp227 (Supplementary Fig. 2b). The Mg^2+^ was bound to protein in M2 site predominantly through water molecules and Asp195 (Supplementary Fig. 2b). However, no electron density for either P_i_ or PP_i_ was observed in the TgPPase active site despite addition of PP_i_ during crystallization. Therefore, it is feasible that the observed conformation in the active site represents a state where both P_i_s have already dissociated.

Based on cues from crystal structures of these apicomplexan PPases, we tested the functional importance of selected active site residues by measuring enzyme kinetics of wild type and mutant PfPPase. We generated point mutations of three Asp residues (Asp198, Asp203 and Asp235) to Asn residues along with conversions of Lys136 and Arg158 to Arg and Lys respectively. As noted from the kinetic parameters calculated at 1.4 nM PfPPase enzyme concentration, the D235N and D198N perturbed activities modestly (~5 fold and ~6 fold reduction in k_cat_, respectively) and therefore may not play major catalytic roles (Table 5). In contrast, enzymatic activity of D203N was ~600 fold lower at aforementioned PfPPase enzyme concentration, suggesting its major catalytic role in PP_i_ hydrolysis (Table 5). These results are in agreement with previous findings on ScPPase^29, 30^. Comparison of PfPPase with TgPPase suggested that Asp203 was a structural counterpart of Asp195 in TgPPase where TgPPase Asp195 simultaneously co-ordinates M1 and M2 (Supplementary Figure 2b). It is known that two Mg^2+^ ions in M1 and M2 site bridge a water molecule between them, generating the reaction nucleophile^29^. Therefore, D203N mutation might have impaired Mg^2+^ binding to PfPPase that thus perturbed the nucleophile generation and hence the hydrolysis rate.

**Table 5:** Kinetic parameters of PP_i_ hydrolysis for mutant and wild type PfPPase.

Protein	k_cat_(s^−1^)	K_m_(µM)	k_cat_/K_m_ (M^−1^s^−1^)
Wild Type	266 ± 11.4	10 ± 1.8	2.66 × 10^7^
D235N	52.5 ± 3.4	45.7 ± 2.1	1.16 × 10^6^
D198N	25.8 ± 5.3	42.6 ± 2.1	1.65 × 10^6^
D203N	0.53 ± 0.02	53.8 ± 8.3	9.85 × 10^3^
K136R	21.8 ± 4.6	402.8 ± 16.4	0.53 × 10^5^
R158K	60.4 ± 6.6	64.2 ± 3.2	0.94 × 10^6^

In contrast to Asp mutants, the K136R variant of PfPPase showed a drastic 40-fold increase in K_m_ indicating an overall compromise in affinity for PP_i_, along with 10 fold reduction in catalytic turnover of PP_i_ hydrolysis (Table 5). By contrast, R158K was less marked and only modestly perturbed the k_cat_ and K_m_ (Table 5). These results suggest possible loss of favorable interactions between mutant residues and PP_i_, which thus impair the catalytic rate by unfavorable positioning of electrophilic P_i_ in the P2 site with respect to the catalytic water.

Thus, mutational probation of active site of PfPPase confirms the functional relevance of the active site residues. Given the fact that PfPPase and yeast PPase active sites are identical the published catalysis model is applicable to PfPPase as well^29–31^.

### Dimeric crystal structures of PfPPase and TgPPase

In the asymmetric unit of PfPPase crystals, four chains formed two non-crystallographic dimers, and dimerization of the fifth monomer was mediated via the crystallographic 2-fold axis. This dimeric crystal structure of PfPPase was consistent with our gel filtration and BN-PAGE data, though gel filtration also suggested a small fraction of tetrameric form that was not observed in the crystal structure. The dimensions of the observed PfPPase dimer are ~98 Å × 48 Å × 58 Å and it is arranged such that the α_5_ helices in the two monomers lie anti-parallel to each other (Fig. 5a). The area for buried surfaces at dimer interfaces is ~ 2650 Å^2^ per subunit. There are two distinct dimer interfaces that lie opposite to each other in the assembly - one face has the strap-like PfPPase N-region (residues 36–69), whereas the other has α_3_ helix (Fig. 5a). In the N-region, α_1_ helices of each subunit together form a cluster of hydrophobic residues (Fig. 5b). Interfacial interactions that exists at this hydrophobic patch are as follows; (1) aliphatic-aliphatic hydrophobic interactions between Ile55 and Ile59 (ii) aliphatic-aromatic interactions between Ile55 and Tyr160, and (iii) π-π stacking force between Phe50 (Fig. 5b). This hydrophic patch is stabilized by a network of hydrogen bonds formed between asparagines [a cluster of residues rich in asparagines (Asn49, Asn52-54, Asn58) and Asn62] of N-region with PPase domain (Fig. 5b and c). All interfacial asparagines have self evident electron densities and all interfacial residues are highly conserved and but limited to the Plasmodium species only (Fig. 5d and e). This N-region contributes ~1800 Å^2^ to average buried surface area at interface, and this data supports its consideration as a physiologically relevant interface. The second interface engages α_3_ helix of the PPase domain from each subunit (Fig. 5e). The side chain interactions that stabilize this interface are hydrogen bonds between Ser266 and Glu270 and NH…π between Arg274 and His263 (Fig. 5f). Like PfPPase, TgPPase also formed dimers in solution and in the crystal (Supplementary Figure 1b). Two TgPPase subunits buried 1780 Å^2^ (50%) of total ASA and are held together mainly by a network hydrogen bonds and Van der Waal forces. Some notable interactions include salt bridges between Arg254 and Glu282 along with edge to face π-π stacking interactions between Trp164 and Trp266 (Supplementary Figure 1c).

### Comparison of Eukaryotic Family I PPases reveal diversity in dimerization modes

**Figure 6:**
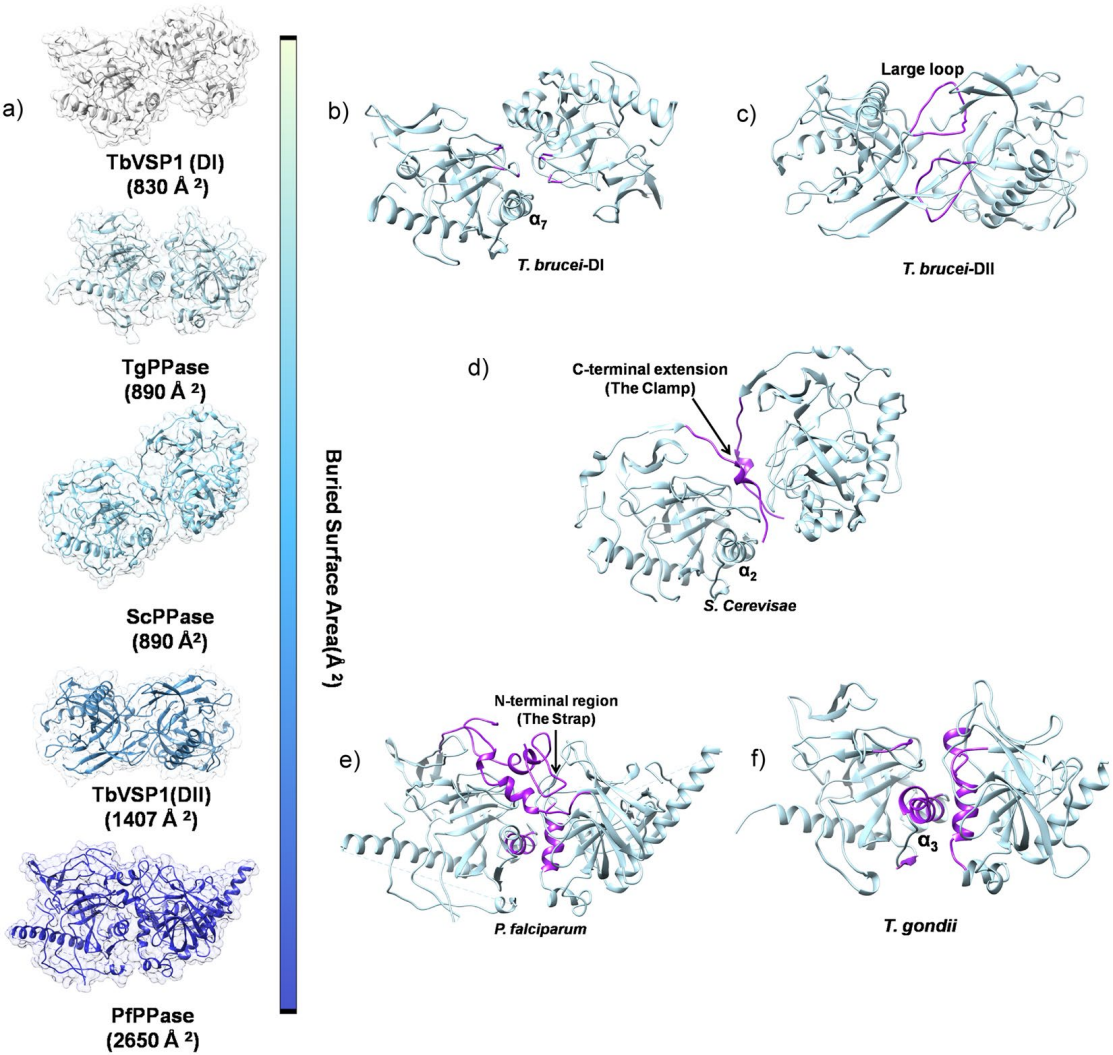
Diverse modes of interactions in eukaryotic PPases. (**a**) Surface representations of dimeric PPases with crystal structure information in ascending order of average buried surface areas. (**b**–**f**) Oligomeric crystal structures of eukaryotic PPases. The structural elements forming monomer-monomer interfaces are highlighted in purple.

**Figure 7:**
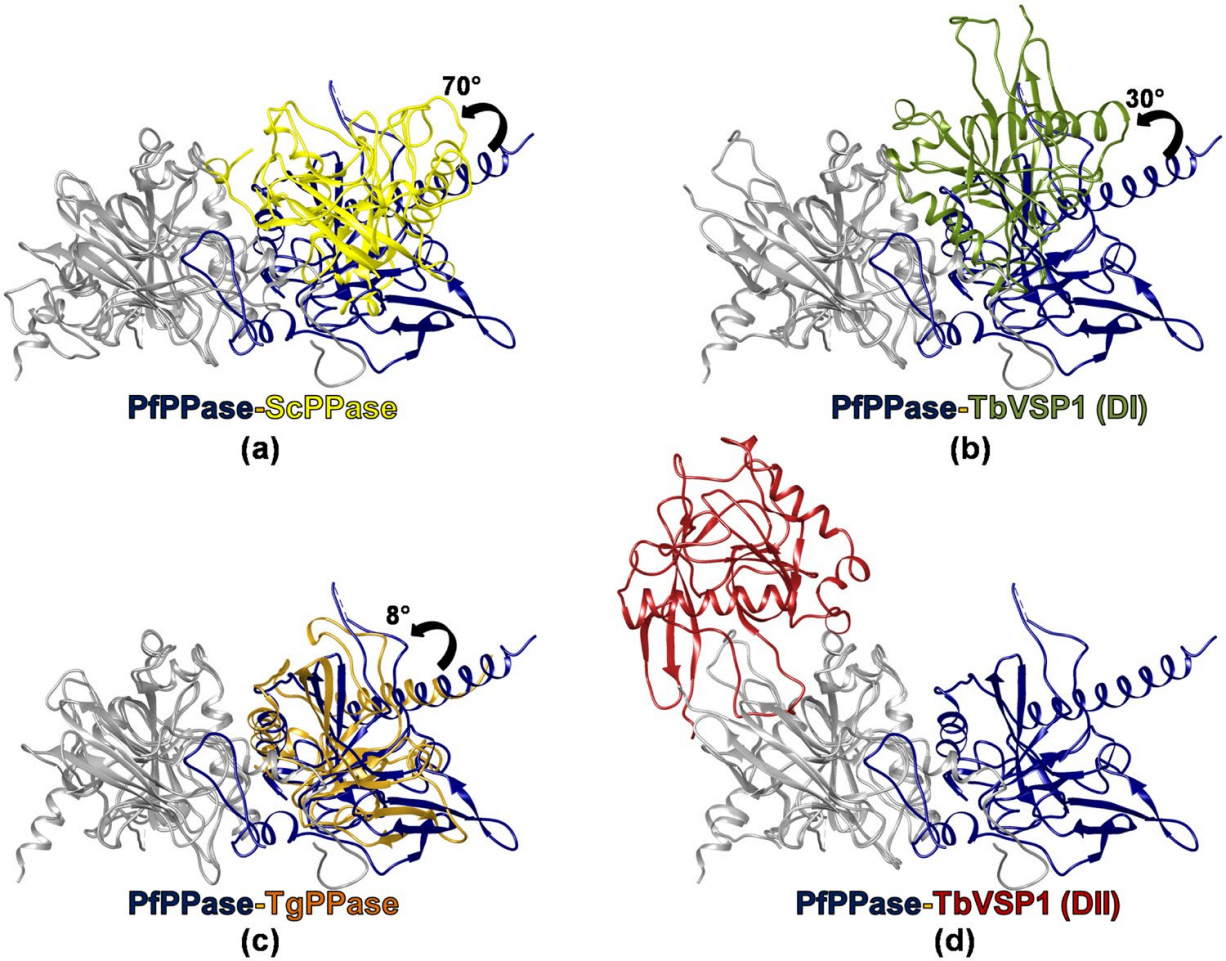
Different modes of dimerization revealed by rotational differences. (**a**–**d**) TgPPase dimer and dimers of ScPPase, TbVSP1 superposed onto a subunit of PfPPase. Position of fixed subunits of PPases are colored in white, whereas rotationally different subunits were colored as follows; PfPPase (blue), TbVSP1-DI (olive green), TgPPase (orange) and TbVSP1-DII (red).

**Figure 8:**
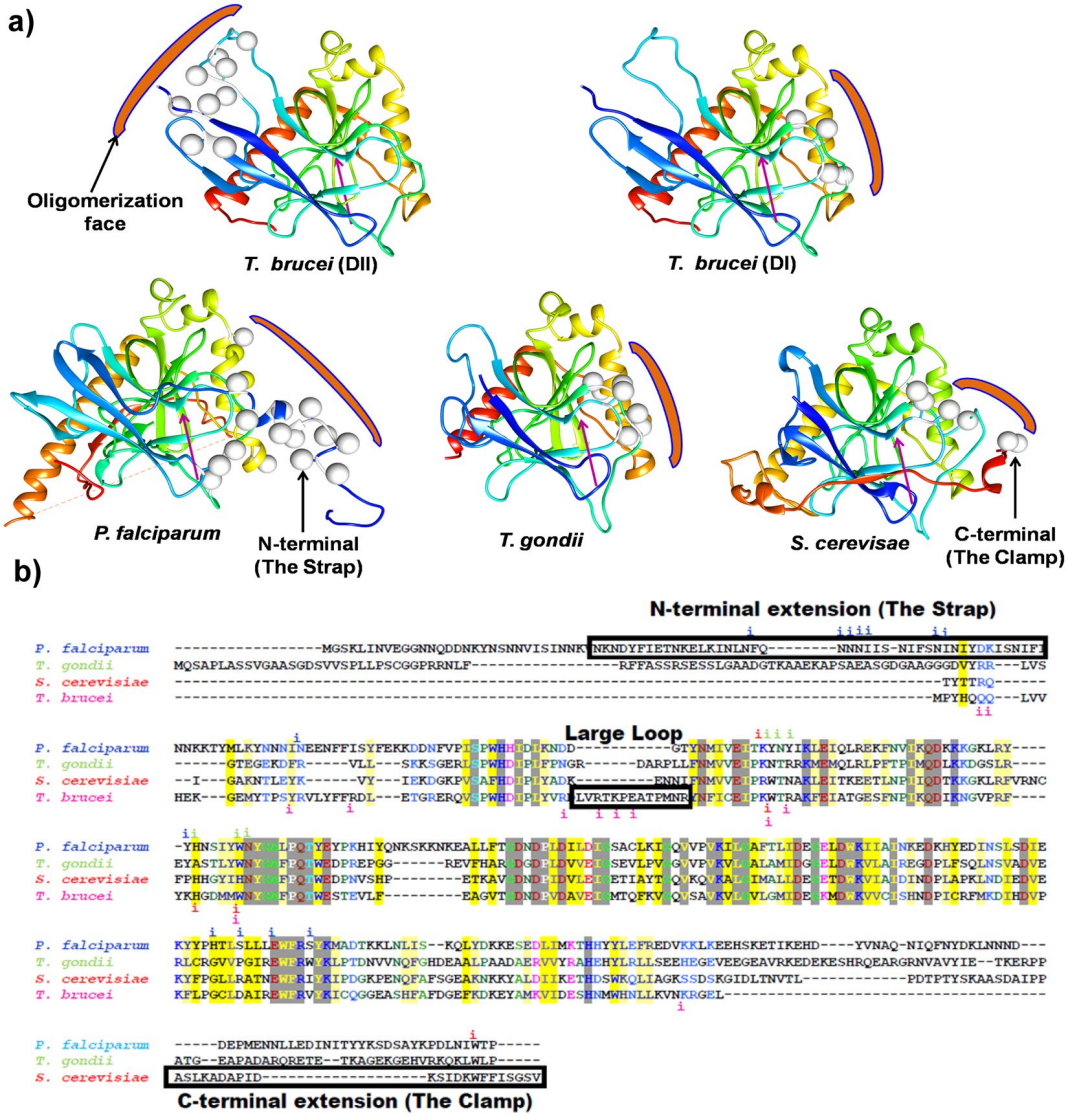
Strategies of diversification in oligomerization modes. (**a**) Crystal structures of PPase monomers colored in rainbow color (monomer’s spectrum runs through blue/cyan to red i. e. from N-terminus to C-terminus) showing oligomerization faces (orange bracket). Interfacial residues are mapped as spheres onto the structure at the equivalent C_α_ residues and orientation of active site is indicated by purple arrow. (**b**) Sequence alignment of eukaryotic PPases where crystal structures are known. The interfacial residues (i) colored according to the species are mapped on to the sequence from structural data; specific and unique structural elements are boxed.

Previously, the oligomeric assembly of eukaryotic family I PPases was reported to be mostly dimeric before our analysis of TbVSP1 showed a tetrameric arrangement^21^ (dimer of dimers). Interestingly, the thus far studied dimeric PPases are different from each other in their monomer-monomer contacts as revealed by their buried surface areas; amongst the known set from PPase structures in PDB the PfPPase forms the tightest dimer as judged by PISA analysis (Fig. 6a). This prompted us to analyze the underlying structural features responsible for the disparity atomic embraces displayed by dimeric PPases. In the crystals of TbVSP1, each monomer packs against two other monomers having two different interfaces^21^. The small dimer DI (830 Å^2^) forms via loops between strands β_6_-β_7_ and β_4_-β_5_^21^. The larger dimer-DII (1407 Å^2^) forms mainly via extensive contacts between a “long loop” that connects β_8_-β_9_ and residues from β_1_ and β_7_ of the PPase domain^21^ (Fig. 6b and c). By contrast, crystal structure of dimeric ScPPase (930 Å^2^) reveals that its monomers are held by their C-terminal extensions, which are important for stability of the dimer^21, 29^ (Fig. 6d). As revealed in previous sections, for PfPPase its N-region and α_5_ helix are two predominant structural elements that assemble the PfPPase dimer (Fig. 6e). On the other hand, TgPPase monomers are joined by α_3_ only (Fig. 6f). From aforementioned structural comparisons, it is clear that Pf and TgPPase dimerize differently. Further, there is strong variance in how TbVSP1 (α_7_) and ScPPase (α_4_) associate (Fig. 6b,c and d). In order to visualize these different modes of PPase dimerizations, we superimposed the dimeric structures of PPases in a way that the position for one monomer is fixed (Fig. 7a–d). PfPPase was used as reference dimer and rotational differences suggested that position of second monomer of TbVSP1 (DI) and ScPPase varied considerably, while the dimerization modes of PfPPase and TgPPase are more similar (Fig. 7c). Strikingly, TbVSP1 (DII) showed different spatial position of the second monomer (Fig. 7d). This could be attributed to the observation that the oligomerization face of DII-TbVSP1 is on the opposite face of PPase domain with respect to the active site, as opposed to in other PPases, which have it on the same face as the active site (Fig. 8a). The differences in subunit orientation in Tb, Tg, Sc and Pf PPase dimers are likely to originate from the diversity in sequence of amino acids contributing to the dimerization interfaces (Fig. 8b). It is evident from the sequence alignments that there are very few overlapping (conserved) interfacial residues (Fig. 8b). Dissimilar modes of association could also be attributed to specific secondary structure elements that are involved in dimerization (Fig. 8b). For example, a long loop (residues 198–223) is present in TbVSP1 and is conserved across kinetoplastida, however, TbVSP1 lacks the C-terminal extension of fungal PPases^21^. Similarly, a strap-like N-region extension seems limited to *Plasmodia*. Although a C-terminal extension similar to ScPPase is also present in the primary sequence of PfPPase and TgPPase, it is not involved in dimer formation of either of them, as revealed by their crystal structures and gel filtration analysis (Fig. 8b and Supplementary Figure 4). Therefore, comparative analyses of PPase crystal structures shows that there is little consensus in the modes of dimer formation across these eukaryotic family I soluble PPases. The functional roles of this structural diversity on PPase function, if any, are not apparent and further studies may address this.

## Conclusions

Although the canonical domain architectures of eukaryotic soluble PPases seems well conserved, there is increasing structural evidence for divergence in their oligomeric assemblies via gain, loss or extension of N/C terminal regions in their sequences. Here we have demonstrated that the N-region of PfPPase is indispensable for enzyme stability and oligomeric integrity. We have compared and contrasted crystal structures of apicomplexan, yeast and kinetoplastid PPases. We also reveal a general heterogeneity in the dimerization modes of these eukaryotic PPases. This study thus provides a detailed architectural glimpse of apicomplexan PPases, and we hope that it will be useful in supporting future studies on phosphate metabolism pathways in these parasites.

## Methods

### Production of PfPPase and TgPPases

The ORFs of full length PfPPase (residues 1–380) were cloned in pETM41 using *NcoI* and *KpnI* restriction sites. Transformed *E*. *coli* BL21-CodonPlus was grown in LB medium containing 50 μg/ml kanamycin to an OD_600_ of 0.6–0.8 at 37 °C. Expression of the recombinant proteins was induced by the addition of 0.5 mM isopropyl β-D-galactoside, and incubation was continued for a further 20 h at 18 °C. The recombinant PfPPase bears a MBP-6X histidine tag. Briefly, bacterial cells were lysed by sonication in a buffer (100 mM HEPES-Na pH 7.2, 500 mM NaCl, 10% glycerol, and 5 mM β-mercaptoethanol) containing protease inhibitor. Affinity purification was performed on amylose resin (NEB) and Ni-NTA (His-Trap FF, GE healthcare) using an AKTA FPLC system. Both tags were cleaved with TEV protease followed by dialysis in low salt buffer (30 mM HEPES pH 7.4, 30 mM NaCl, 1 mM DTT). Protein was subsequently applied to Q-Sepharose (GE healthcare) column for further purification and removal of TEV protease. Finally, pure fractions were pooled and concentrated to 10 mg ml^−1^ with 10 kDa cutoff centrifugal devices (Millipore) followed by Gel Permeation Chromatography (GPC) on S-200–16/60 column (GE- healthcare) in a buffer containing 30 mM HEPES-Na pH 7.2, 100 mM NaCl and 1 mM DTT. TgPPase was cloned in pETM11 and purified using Ni-NTA chromatography followed by Q-sepharose ion exchange chromatography and GPC.

### Real-Time PCR

Total RNA was extracted from 3D7 *P*. *falciparum* cells using intra-erythrocytic parasite stages and by trizol method. For time-course studies, *P*. *falciparum* parasites were taken at 8–12 h, 24–28 h, 34–42 h and 42–48 h post synchronization representing rings, early trophozoite, late trophozoite/early schizont and mature schizonts - as confirmed by microscopy. Total 1.5 µg of total RNA was amplified in 42 µl reaction volume using oligo dT primers and Superscript III reverse transcription kit (InVitrogen) following which the reaction mix was diluted ten times prior to real-time amplification. To study temporal expression of predicted PfPPase gene, real time PCR was performed on ABI step one plus (Applied Biosystems) using Quantitect SYBER Green I mix (Qiagen, Hamburg, Germany). Threshold cycle (C_t_) values were determined using ABI prism software and 2^−ΔΔCt^ was used to calculate relative expression values. PfSerRS (Seryl- tRNA synthetase) gene was the internal reference control and ΔC_t_ value of the ring stage was used as the calibrator. Primers used for amplification were ~200 bp in size and were first optimized to give maximum amplification. The amplification factor for primers of PfPPase and PfSerRS were 1.96 and 1.98 respectively.

### Measurement of kinetic activity of PfPPase

PfPPase activity was measured based on methods described previously^25^. PfPPase was added to the reaction mixture-carrying varying concentrations of substrates (PP_i_, polyP_3_ and ATP) in reaction buffer suitable for optimum pH with 100 mM NaCl at 37 °C at for 5 min. The optimum pHs for PP_i_, polyP_3_ and ATP hydrolysis were 7.2, 7.0 and 7.2 respectively. To stop enzymatic reactions, one volume of malachite green reagent was mixed with four volumes of enzymatic reaction to be analyzed. The mixture was incubated for 3 min and absorbance at 623 nm was measured with a spectromax UV/VIS spectrophotometer (BIO RAD). To measure activity in total cell lysate, isolated malaria parasite trophozoites were washed with buffer containing 30 mM HEPES pH 7.2, 116 mM NaCl, 5 mM KCl, 5 mM glucose (to prevent premature cell lysis and release of proteases) and resuspended in the same buffer. The cells were broken by sonication (20% amplitude, five, 2 seconds pulses). The activity was also measured from sub-cellular fractions obtained via lysis with 1% Triton-X-100 as described previously^32^. Each data point was produced from individual experiments that were performed in triplicates. Absorbance values were read twice using spectrophotometer to ensure integrity of the data.

### Protein Thermal Shift Assays

These were performed with 2.5 μM PfPPase in 30 mM HEPES-Na, pH 7.2, 100 mM NaCl and a 1× dilution of SYPRO orange dye (Invitrogen). The dye was excited at 490 nm and emission light was recorded at 575 nm while the temperature was increased in increments of 1 °C per minute from 20–98 °C. Control assays were carried out in the absence of protein or dye to ensure that no fluorescence signal was recorded. Thermal shift experiments of PfPPase complexes were performed using analogs Imidodiphosphate (PNP) (Sigma Aldrich) and AMPPNP (Sigma Aldrich). These are chemical mimics of PP_i_ and ATP respectively. Both AMPPNP was used at 5 mM concentration and mixed with 1 mM of each Mg^2+^, Co^2+^, Zn^2+^ and Mg^2+^.

### Immunolocalization and western blotting

Immunofluorescence assays were performed using protocols described previously^24^. Purified primary rabbit anti-PfPPase antibody at 1:200 dilution was used. Secondary antibodies used were Alexa flor 545 (Invitrogen) and Alexa flor 595 (Invitrogen). Mitotracker Red CH_2_X ROS was used to stain the parasite mitochondria. For western blotting experiments, proteins were separated on SDS-PAGE gel and analysis was performed using anti-PfPPase (1:500). Rabbit Anti- PfNAPL (1:2000) antibodies were used as internal control^33^.

### Crystallization of PfPPase and TgPPases

A single peak corresponding to dimer of PfPPase was collected from GPC. This protein solution contained 3 mM MgCl_2_ and 1 mM PP_i_ (Sigma Aldrich) and was used for co-crystallization (10 mg ml^−1^). Crystallization conditions were initially sought by vapor diffusion method at 293 K using commercially available crystallization screens. Crystals of seleno-methionine substituted PfPPase were obtained in buffer condition 8% Tacsimate pH 8.0 and 20% PEG3350. Multiple single crystals were obtained for the protein in drops ~4 days. PfPPase crystals were cryo-protected with 18% ethylene glycol in mother liquor prior to mounting. TgPPase (concentrated to 10 mg ml^−1^) crystals were obtained within six to eight days in MORPHEUS crystallization screen (Molecular Dimensions). The well condition corresponded to 10% PEG4000, 20% glycerol, 0.03 M glycols and 0.1 M HEPES/MOPS pH7.5.

### Structure determinations and refinements

Selenomethionine (Se-Met)-labeled PfPPase crystals were protected by a cryoprotectant containing 8% Tacsimate pH 8.0 and 20% PEG3350, and the data were collected at BM14, ESRF, Grenoble at the peak wavelength of 0.9762 Å at 100 K. The dataset was indexed, integrated and scaled using HKL2000^34^. The Se-Met crystals of PfPPase belonged to monoclinic space group C2 with cell dimensions of a = 253.19, b = 85.23, c = 108.45 Å with five molecules in the asymmetric unit (ASU). 22 Selenium were identified in the ASU using HySS^35^ and subsequent phasing was done using Autosol^36^. Autosol derived phases were used to build a partial model with AutoBuild^37^. The complete model was built using Coot^38^ and the model was refined with phenix.refine^39^.

Native data were collected for TgPPase at BM14, ESRF, Grenoble. TgPPase crystal structure was determined by molecular replacement method with the program PHASER^40^ within the PHENIX suite, using yeast soluble PPase as the search model (PDB code = 1WGJ), with 44% sequence identity from residue 107 to 304 of the target. The results from molecular replacement for TgPPase showed a translation function Z-score of 15 that strongly suggested a correct solution. The atomic positions obtained from molecular replacement and the resulting electron density maps were used to build (AutoBuild and Coot) the TgPPase structures and initiate crystallographic refinement (phenix.refine). The coordinates and structure factors for PfPPase and TgPPase have been deposited in the PDB under accession code 5WRU and 5WRT respectively. Structure figures were generated using CHIMERA^41^ & PyMOL (Schrodinger).

## Electronic supplementary material

Supplementary information.
