# Supplementary material for: Structural and Biochemical Characterization of Apicomplexan Inorganic Pyrophosphatases
Source: Sci Rep. 2017 Jul 12;7:5255. doi: 10.1038/s41598-017-05234-y (PMC5507929; doi:10.1038/s41598-017-05234-y)
Supplement: Supplementary file 1 — Supplementary information. [file 41598_2017_5234_MOESM1_ESM.doc]

**Structural and Biochemical Characterization of Apicomplexan Inorganic Pyrophosphatases**

**Abhishek Jamwal^1, 2^, Manickam Yogavel^1^, Malik. Z. Abdin^2^, Swatantra. K. Jain^2, 3^and Amit Sharma*^1^**

^1^Molecular Medicine Group, International Centre for Genetic Engineering and Biotechnology, Aruna Asaf Ali Marg, New Delhi, 110067, India

^2^Department of Biotechnology, Jamia Hamdard, New Delhi, 110063, India

^3^Department of Biochemistry, Hamdard Institute of Medical Sciences, 110063, New Delhi, India

*****Corresponding Author, E-mail ; amit.icgeb@gmail.com


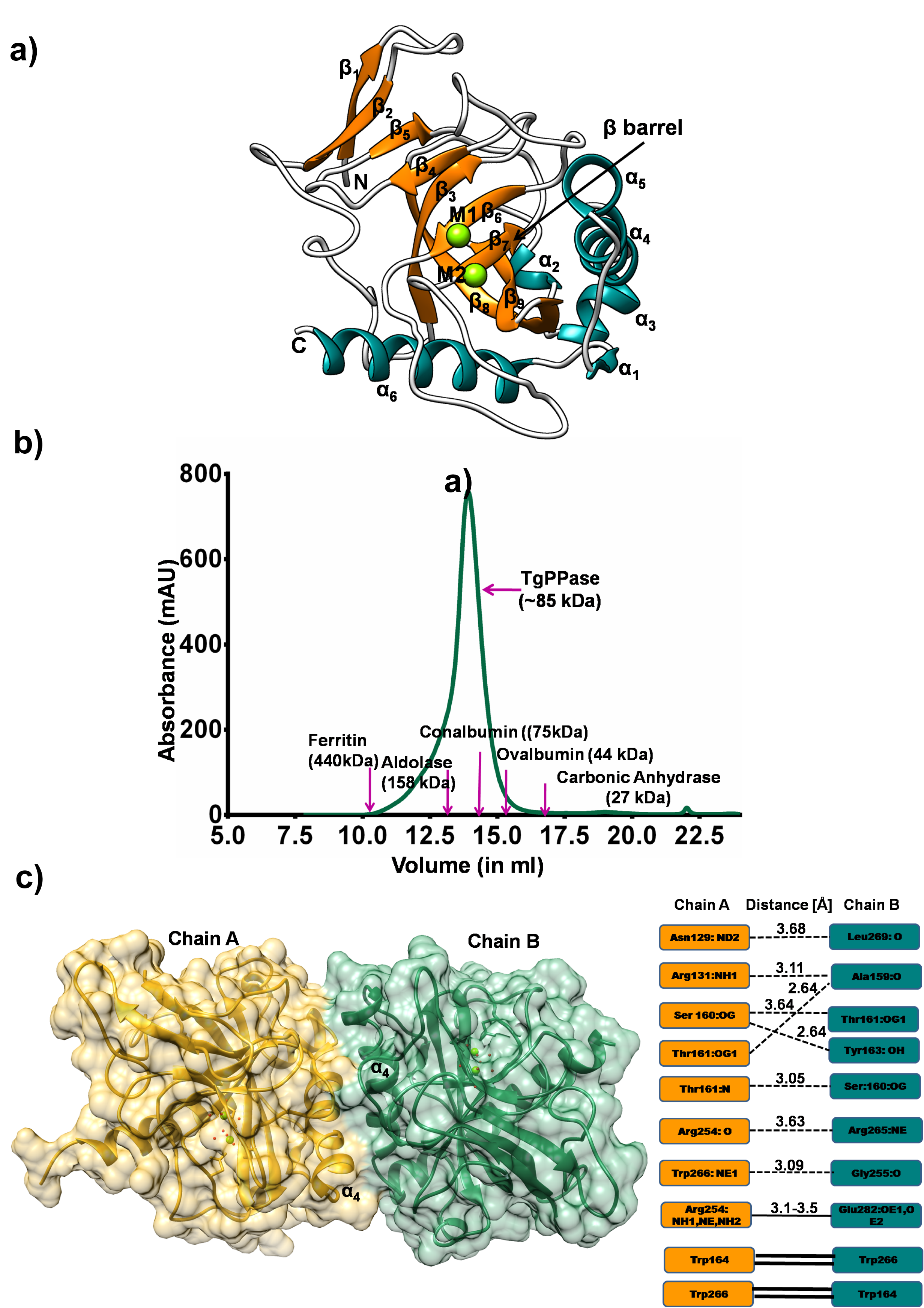


**Figure Supplementary 1. Crystal structure of TgPPase. a)** Cartoon representation of TgPPase structure, b) Size exclusion chromatography elution profile/peaks of full-length TgPPase, monitored by absorbance at 280 nm. Standard molecular weight markers are indicated by green arrows on elution volume axis, c) Right panel shows hydrogen bonds and salt bridges as solid dashed lines, whereas hydrophobic stacking interactions are shown as thick solid lines.


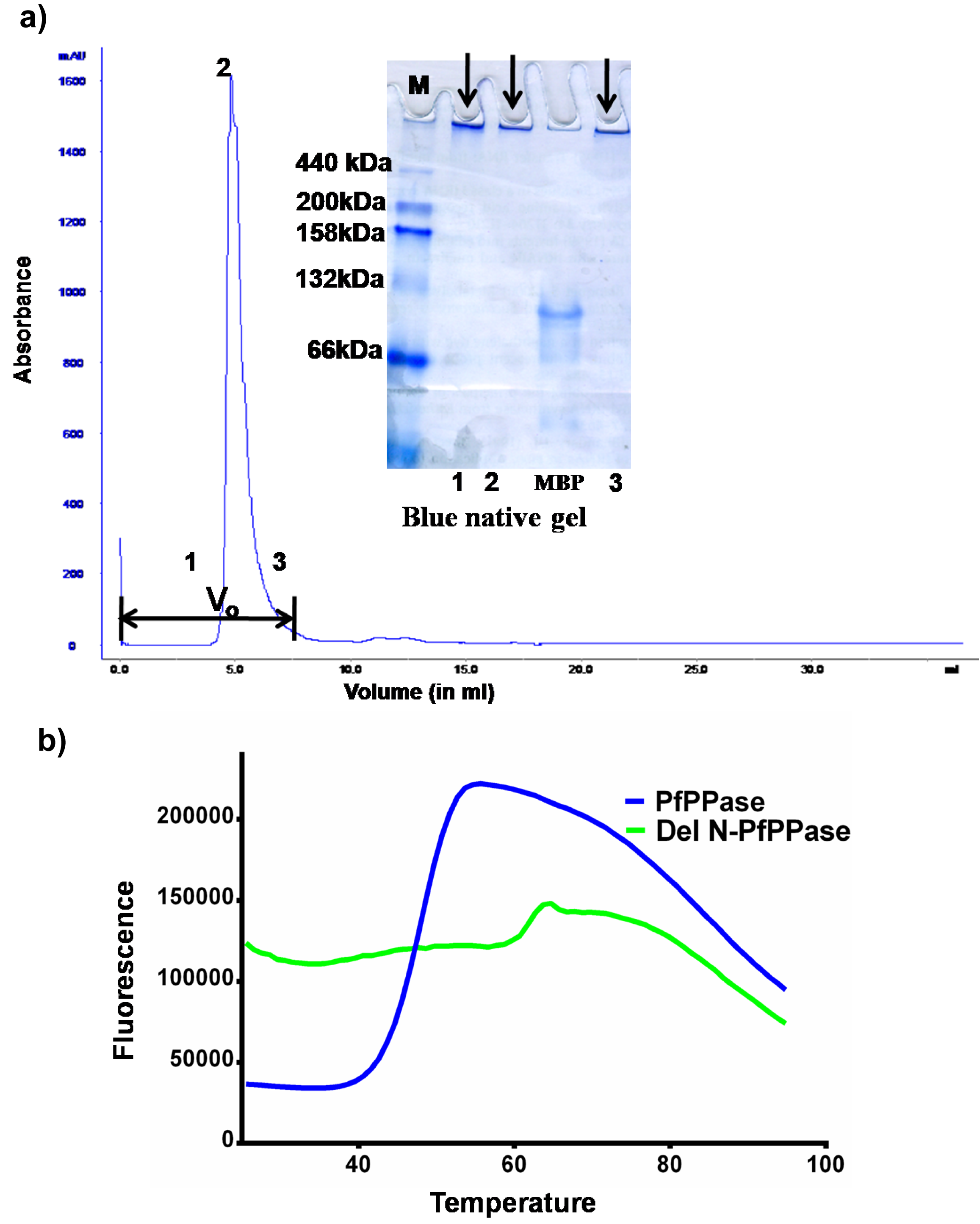


**Supplementary figure 2. N-terminal extension affects PfPPase folding,** a) Gel filtration profile of delN-PfPPase showing its elution in void volume which is indicative of high molecular weight aggregation. A 10 % blue native-PAGE with eluted fractions 1, 2 and 3 that are trapped in wells suggesting that del-N-PfPPase is aggregated; b) Thermal stability of ΔN-PfPPase could not be derived since the fluorescent probe bound to the protein at room temperature, another indication that the protein was not properly folded.


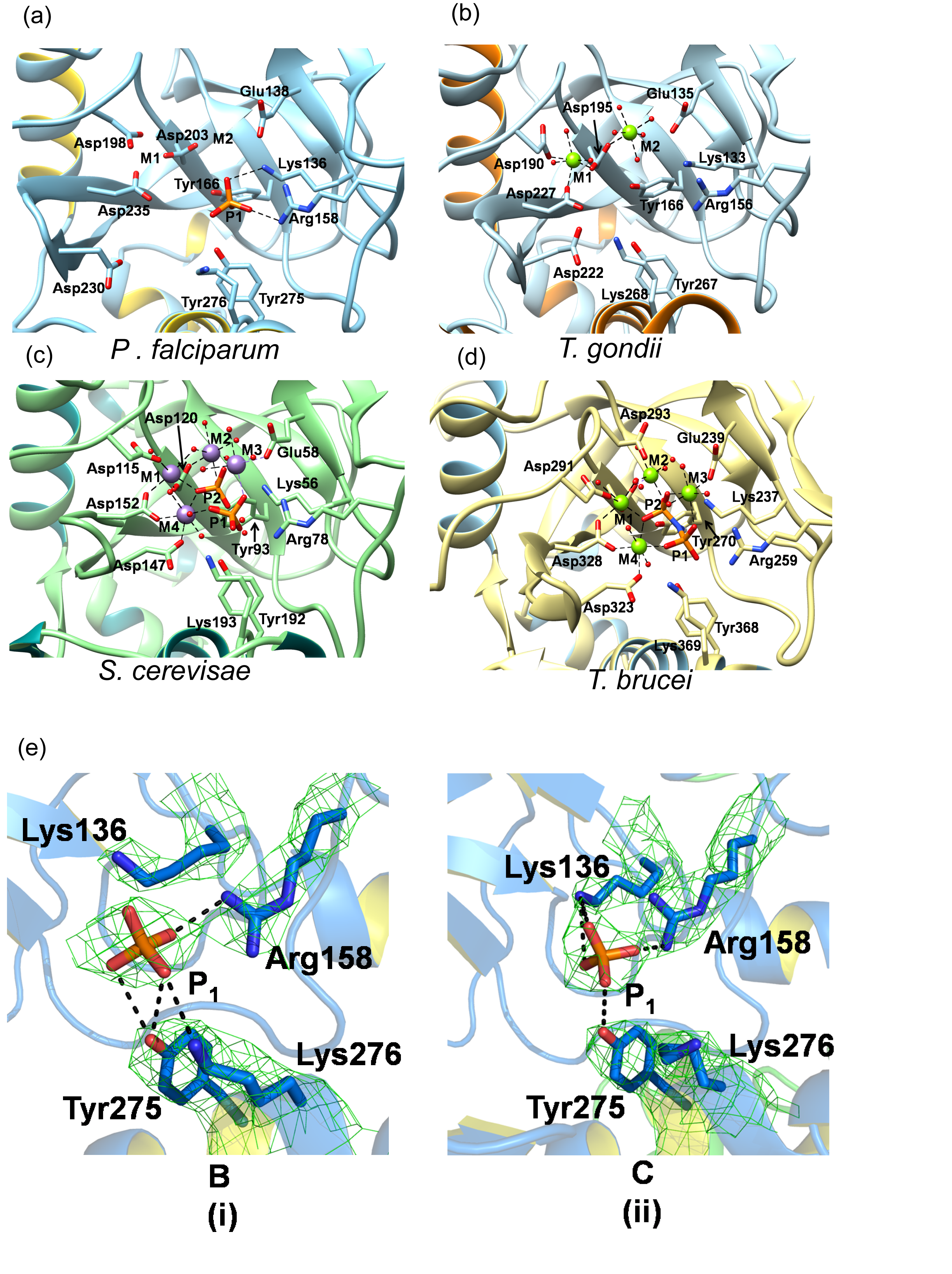


**Supplementary Figure 3**. Cartoons showing active sites of (a) PfPPase (b), TgPPase (c) ScPPase and (d)TbVSP1. Side chains are shown as sticks and metal ions Mg^+2^ (chartreuse) and Mn^+2^ (purple) are shown as spheres . (e) Stereo view of active site of subunits showing 2Fo-Fc maps of electron densities for a phosphate (P1) molecule and its surrounding residues. Hydrogen bonds between P1 and amino acid side chains dashed lines.


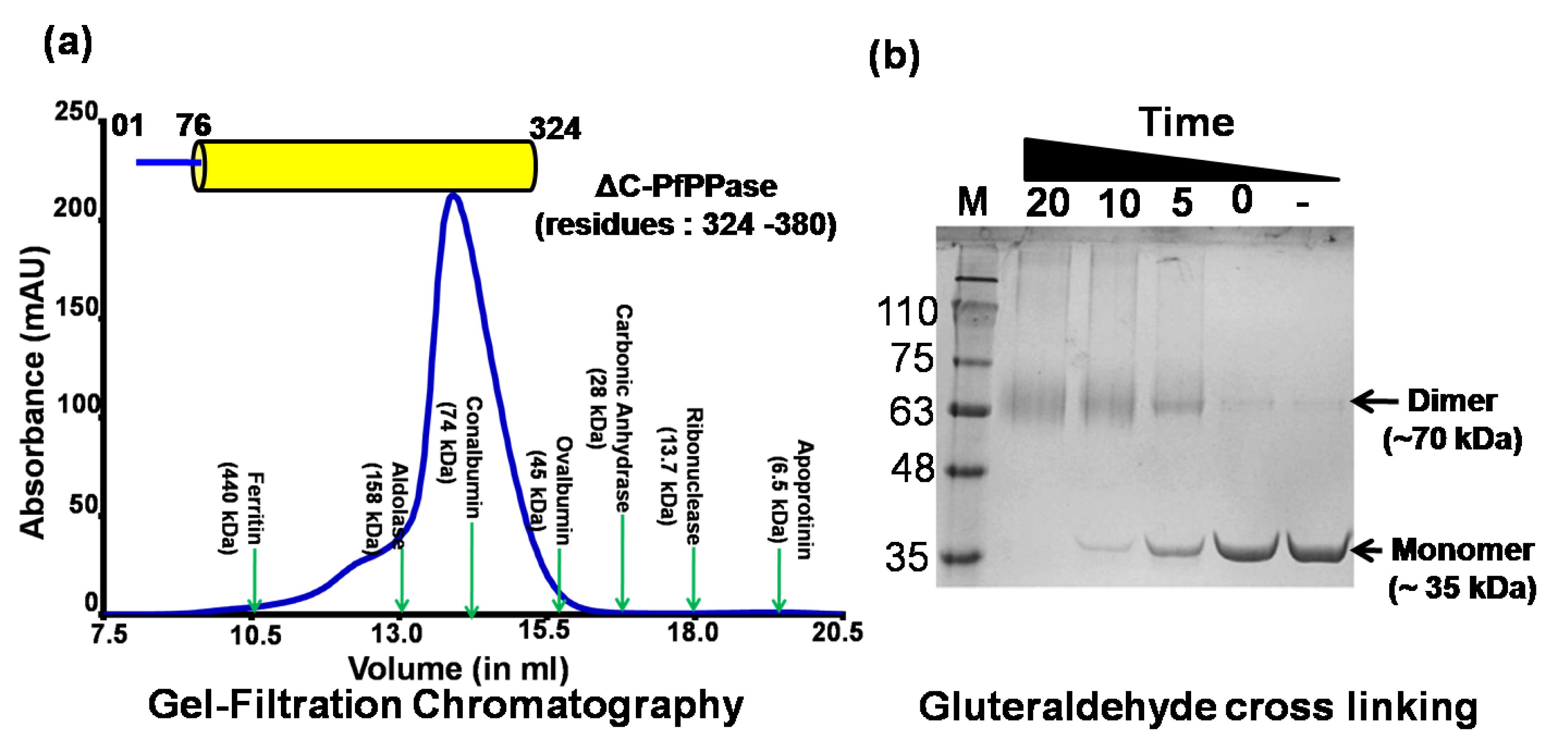


**Supplementary Figure 4. (a)** Left panel shows size exclusion chromatography trace for ΔC-PfPPase. The observed molecular mass estimate ~ 70 kDa. (b) depicts Gluteraldehyde cross linking gel with a major band observed near 70 kDa corresponding to a dimer.
